# A Case and a Question

**Published:** 1874-11

**Authors:** Jno. W. Booth

**Affiliations:** Tally-Ho, North Carolina


### A CASE AND A QUESTION.

BY JNO. W. BOOTH, M.D., OF TALLY-HO, NORTH CAROLINA.

In the Spring of 1866, I was consulted by Mr. H. S. G. about his wife. He stated that she had accompanied him on horseback to a store some seven miles distant, being, as she thought, about four months advanced in her fourth pregnancy. While there she withdrew to obey a call of nature, and in the act discharged *his hat full of cold blood* (coagula) from her vagina, without any pain. Making due allowance for the exaggeration that is common with the unprofessional when they estimate blood, I supposed that she had lost a good deal. Inasmuch as Mrs. G.'s labors previously had been very rapid, not lasting half an hour after the first evidence perceived by her, I supposed that she had aborted connecting labors, the first stage of which, or most of it, must have been painless, with a painless abortion.

I had been taught that when any considerable quantity (a tablespoonful) of blood is lost from the uterus in the early months of pregnancy, (*Meig's Woman and Her Diseases*) abortion is inevitable, and had not then seen any thing to shake my confidence, in a very high authority, on this subject.

I therefore told Mr. G. that if his wife was pregnant, she either had miscarried or would miscarry soon, and advised perfect rest. I do not know whether she followed my directions or not, but on the evening of the 14th of September, I was hastily summoned to Mrs. G., and before I could run my mare, a very fleet one, two miles, she had been delivered of a healthy, well-developed male child, of good average size, it being some five months after the attack of hemorrhage.

This case furnished food for much reflection, and had proved to my mind that abortion may be simulated, I not being aware that the same had happened to any body else, till I saw something similar in the *Journal of the Gynœcological Society*, of Boston, in September, 1869. I have since seen a case or two somewhat similar. For instance, I treated a woman for severe uterine hemorrhage, and in five or six months she was delivered of a child, evidently at full term, etc. Was this a case in which a woman was pregnant of twins, and one was thrown off?—or was it simple hemorrhage? If simple hemorrhage, where did the blood come from?

*An Abnormity.*—On the 27th of April, 1872, Sprig Russell, a negro, requested me to visit his wife, saying that she had been delivered of a dead child; that there was no naval-string, and that her mother, who was a midwife, could not find any after-birth.

The parturient was a full-blooded African, rather under medium size, and had been married four months. This was her second pregnancy. Before making an examination of the mother, I thought best to inspect the fœtus.

From the size of head and shoulders, I supposed it to be a six or seven months' child. The hands were over the chest and the legs on the abdomen, as is usual in intranterine life, but the placenta was firmly attached, anteriorly to the child, from the buttocks, over the whole abdomen and chest, throat and lower face, as far as the nose. The placenta was so firmly adherent, that it could not be pealed off with the fingers, and it covered nearly the whole of the arms and legs. The limbs, as far as they could be seen, were under the skin.

This woman has been in bad health, and has not been pregnant since, probably laboring under some uterine trouble.

